# Inhibition of PI3K/mTOR Overcomes Nilotinib Resistance in BCR-ABL1 Positive Leukemia Cells through Translational Down-Regulation of MDM2

**DOI:** 10.1371/journal.pone.0083510

**Published:** 2013-12-11

**Authors:** Jie Ding, Julia Romani, Margarete Zaborski, Roderick A. F. MacLeod, Stefan Nagel, Hans G. Drexler, Hilmar Quentmeier

**Affiliations:** Leibniz-Institute DSMZ-German Collection of Microorganisms and Cell Cultures, Department of Human and Animal Cell Lines, Braunschweig, Germany; Emory University, United States of America

## Abstract

Chronic myeloid leukemia (CML) is a cytogenetic disorder resulting from formation of the Philadelphia chromosome (Ph), that is, the t(9;22) chromosomal translocation and the formation of the BCR-ABL1 fusion protein. Tyrosine kinase inhibitors (TKI), such as imatinib and nilotinib, have emerged as leading compounds with which to treat CML. t(9;22) is not restricted to CML, 20-30% of acute lymphoblastic leukemia (ALL) cases also carry the Ph. However, TKIs are not as effective in the treatment of Ph+ ALL as in CML. In this study, the Ph+ cell lines JURL-MK2 and SUP-B15 were used to investigate TKI resistance mechanisms and the sensitization of Ph+ tumor cells to TKI treatment. The annexin V/PI (propidium iodide) assay revealed that nilotinib induced apoptosis in JURL-MK2 cells, but not in SUP-B15 cells. Since there was no mutation in the tyrosine kinase domain of BCR-ABL1 in cell line SUP-B15, the cells were not generally unresponsive to TKI, as evidenced by dephosphorylation of the BCR-ABL1 downstream targets, Crk-like protein (CrkL) and Grb-associated binder-2 (GAB2). Resistance to apoptosis after nilotinib treatment was accompanied by the constitutive and nilotinib unresponsive activation of the phosphoinositide 3-kinase (PI3K) pathway. Treatment of SUP-B15 cells with the dual PI3K/mammalian target of rapamycin (mTOR) inhibitor BEZ235 alone induced apoptosis in a low percentage of cells, while combining nilotinib and BEZ235 led to a synergistic effect. The main role of PI3K/mTOR inhibitor BEZ235 and the reason for apoptosis in the nilotinib-resistant cells was the block of the translational machinery, leading to the rapid downregulation of the anti-apoptotic protein MDM2 (human homolog of the murine double minute-2). These findings highlight MDM2 as a potential therapeutic target to increase TKI-mediated apoptosis and imply that the combination of PI3K/mTOR inhibitor and TKI might form a novel strategy to combat TKI-resistant BCR-ABL1 positive leukemia.

## Introduction

Expression of the Philadelphia chromosome (Ph), i.e. the t(9;22) chromosomal translocation and the formation of the BCR-ABL1 fusion protein, is the hallmark of chronic myeloid leukemia (CML). BCR-ABL1 is not only present in CML patients, but also occurs in 20-30% of acute lymphoblastic leukemia (ALL) cases. Nilotinib (AMN107) is an effective secondary generation tyrosine kinase inhibitor (TKI) interacting with the ATP-binding site of BCR-ABL1. Compared to the first generation TKI imatinib, nilotinib not only shows a low IC50 value (IC50 20-60 nM vs. IC50 120-470 nM), but also acts against most imatinib-unresponsive BCR-ABL1 mutation variants [1,2]. In phase II clinical trials, nilotinib proved safe and effective for long-term use in CML patients who were intolerant of or resistant to imatinib [3]. Although successful hematologic and cytogenetic responses have been obtained in the vast majority of nilotinib-treated patients, cases showing resistance to nilotinib have been observed [4,5]. Several causes of nilotinib resistance have been described: T315I mutation in the kinase domain of BCR-ABL1 [6-8], overexpression of BCR-ABL1 itself or overexpression of multidrug resistance protein 1 (MDR1) or the Src kinase [9] and down-regulation of apoptotic BAX and CERS1 (ceramide synthase 1) [10]. We previously reported that TKI-resistant cells were not generally unresponsive to TKI, as evidenced by dephosphorylation of the BCR-ABL1 downstream target signal transducer and activator of transcription 5 (STAT5) and extracellular-signal-regulated kinase (ERK). It turned out that BCR-ABL1-independent phosphatidylinositide 3 kinase (PI3K) activation caused the TKI resistance [11]. 

In this study, we set out to dissect the PI3K/AKT/mammalian target of rapamycin (mTOR) pathway to investigate TKI resistance mechanisms and sensitization of Ph+ tumor cells to TKI treatment. Two members of the PI3K/AKT pathway were overexpressed in TKI-resistant cells, GAB2 (Grb-associated binder-2) and MDM2 (human homolog of the murine double minute-2), which stood out as plausible causes for TKI resistance.

GAB2 is a critical signal transducer of BCR-ABL1, which couples growth factor and cytokine receptors to downstream effectors, such as PI3K/AKT/mTOR. Persistent phosphorylation of GAB2 Y452, a PI3K recruitment site, confers GAB2-mediated TKI resistance, whereas GAB2 knockdown or haploinsufficiency increases TKI sensitivity [12]. The PI3K/AKT/mTOR pathway is important for cell survival, proliferation and metabolism [13]. Upon PI3K stimulation, the serine/threonine-specific protein kinase AKT is phosphorylated, which leads to activation of mTORC1. The substrates of mTORC1 include the ribosomal protein S6 kinase (S6K) and the eukaryotic initiation factor 4E binding proteins (4E-BP1) [14,15]. The PI3K/AKT/mTOR signaling pathway is often constitutively activated in malignancy rendering alterations in this pathway potential therapeutic targets [16-18].

MDM2 is a downstream effector of PI3K/AKT pathway, stabilized by AKT-dependent phosphorylation [19]. Cancer cells with AKT pathway activation are sensitive to MDM2 antagonists, confirming the importance of MDM2 for cell survival. Thus for example, nutlin-3 by inhibiting the interaction between MDM2 and p53, displays anti-proliferative and pro-apoptotic activity in various cancers, including mantle cell lymphoma [20], pediatric ALL cells [21], prostate and lung carcinoma [22,23], and chronic lymphocytic leukemia [24,25]. MDM2 can function as an oncogene by downregulation of p53 [26], or through p53-independent mechanisms which regulate proliferation [27] and apoptosis [28]. MDM2 itself is regulated in two ways as a downstream target of the PI3K/AKT pathway: i) it is phosphorylated by AKT; ii) the MDM2 protein levels are affected by the translational machinery through this pathway. 

BEZ235, a dual PI3K/mTOR inhibitor, reduces PI3K and mTOR activity through competitive binding to the ATP-binding site of these enzymes [29]. BEZ235 has proved effective in various cancers by induction of G1 cell cycle arrest and apoptosis, which has already entered phase II clinical trials [30-33]. In our current study, we report that BEZ235 alone induced apoptosis in a low percentage in nilotinib-resistant BCR-ABL1-positive cells. However, the combination of nilotinib and BEZ235 led to a synergistic effect in these cells. The main role of PI3K/mTOR inhibition and reason for apoptosis in nilotinib-resistant cells was the block of the translational machinery, leading to the rapid downregulation of anti-apoptotic protein MDM2. Therefore, MDM2 appears to be a promising therapeutic target with which to sensitize TKI-resistant BCR-ABL1 positive leukemia cells to TKI-induced apoptosis. Combining PI3K/mTOR and TKI inhibition may prove an effective novel therapeutic strategy in TKI-resistant BCR-ABL1 positive leukemia.

## Materials and Methods

### Human cell lines and treatments

The cell lines applied in this study are all held by the DSMZ - German Collection of Microorganisms and Cell Cultures, Braunschweig, Germany (www.dsmz.de). Detailed references and cultivation protocols have been described previously [34]. Nilotinib and BEZ235 (Novartis, Basel, Switzerland) were dissolved in dimethylsulfoxide (DMSO) and stored at -20 °C as a 10 mM stock. 

### Cell cycle analysis and detection of apoptotic cells

Apoptotic cells were detected and quantified with the annexin-V/PI (propidium iodide) method using the TACS Annexin-V-FITC kit (R&D Systems, Wiesbaden, Germany) according to the manufacturer’s instructions. Binding of fluorescein isothiocyanate-labeled annexin-V and PI staining of the cells were determined by flow cytometry on the FACSCalibur (Becton Dickinson, Heidelberg, Germany). For cell cycle analysis cells were fixed with 70% ethanol (-20°C, 20 min on ice), washed with phosphate-buffered saline, and stained with PI (20 μg/ml). DNA contents of cells were determined by flow cytometry.

### Quantitative real-time PCR analysis

Quantitative PCR was performed on a 7500 Applied Biosystems (Darmstadt, Germany) real-time PCR system using the manufacturer’s protocol. RNA was prepared using the RNeasy Mini kit (Qiagen, Hilden, Germany). For mRNA quantification, reverse transcription was performed using the SuperScript II reverse transcriptase kit (Invitrogen, Karlsruhe, Germany). Expression of genes was assessed using the SYTO-82 PCR Master Mix (Invitrogen) with RPS9 as internal control. Primers were the following: PI3KCA forward: 5’-GGT CTG TAT CCC GAG AAG C-3’; PI3KCA reverse: 5’-GAG GCC AAT CTT TTA CCA AGC-3’; AKT forward: 5’-GCT CTT TGT GAT GGA TGA GGA-3’; AKT reverse: 5’-CTC AGG CTG CCA TCA TCT G-3’; TSC1 forward: 5’-CTT CTT GCC ATG CTG GAC TC-3’; TSC1 reverse: 5’-CCA CGG TCA GAA TTG AGG TT-3’; TSC2 forward: 5’-CAG GTC TGC AGA GGG TAA AC-3’; TSC2 reverse: 5’-TGC GAT TGT TGA GGC CAC ATT-3’; GβL forward: 5’-GAA TGC CTT GGA GGT CAC AC-3’; GβL reverse: 5’-CCA CAG ACG CGA TGT TCT TG-3’; RAPTOR forward: 5’-TTG TGC CTG AAT GTT GGT GTG-3’; RAPTOR reverse: 5’-CGA TGG TTT CCA GAG CTT TCT-3’; RICTOR forward: 5’-GGC AGC TGA GGC AAA AAC TA-3’; RICTOR reverse: 5’-ACG AAT AAA TGC AGA GAG TAT CAG-3’; mTOR forward: 5’-AGT GGG AAG ATC CTG CAC ATT-3’; mTOR reverse: 5’-TGG AAA CTT CTC TCG GGT CAT-3’; P70S6K forward: 5’-TGA GGA TGA GCT GGA GGA G-3’; P70S6K reverse: 5’-GGC CCT CTG TTC ACA CTA G-3’; MDM2 forward: 5’-TGT TGG TGC ACA AAA AGA CAC TT-3’; MDM2 reverse: 5’-GCA CGC CAA ACA AAT CTC CTA-3’; RPS9 forward: 5’-GGG AAG CGG AGC CAA CAT G-3’; RPS9 reverse: 5’-GTT TGT TCC GGA GCC CAT ACT-3’. Relative expression levels were calculated using the ΔΔ Ct-method.

### Western blot analysis

Samples were prepared as described previously [35]. p-mTOR, mTOR, p-S6, S6, p-4E-BP1, 4E-BP1, p-CrkL, CrkL, p-GAB2, CDK4, CDK6, cyclinD1, cyclinD3 and caspase 3 antibodies were purchased from Cell Signalling (New England Biolabs, Frankfurt, Germany). The anti-PARP monoclonal antibody (mAb) was purchased from R&D systems. Anti-GAB2 was obtained from Santa Cruz (Heidelberg, Germany). The anti-GAPDH mAb was purchased from Abcam (Cambridge, UK). MDM2 antibody was from Calbiochem (Darmstadt, Germany). Specific bands on nitrocellulose membranes were visualized with the biotin/streptavidin horseradish peroxidase system (Amersham, Freiburg, Germany) in combination with the “Renaissance Western Blot Chemoluminescence Reagent” protocol (Perkin Elmer, Waltham, MA, USA).

### siRNA transfections

For RNAi studies, siRNAs (small interfering RNA) directed against MDM2 (Hs_MDM2_10 Flexitube siRNA, Qiagen, Hilden, Germany) and GAB2 (Hs_GAB2_1 Flexitube siRNA, Qiagen) were electroporated into cell lines at the final concentration of 20 nM using the EPI-2500 impulse generator (Fischer, Heidelberg, Germany). AllStars Neg. Control siRNA (Qiagen) was used as a negative control. Knockdown efficiency was determined by Western blot. Cells were harvested after 24 h or 48 h, respectively, for further studies.

### Statistical ananlysis

Data were analyzed using the Student’s *t*-test. All experiments reported here represent independent duplicate or triplicate replicates. All data are represented as mean value ± SD and significant differences are indicated as *P<0.05, **P<0.01.

## Results

### SUP-B15 cells are resistant to nilotinib treatment

**Figure 1:**
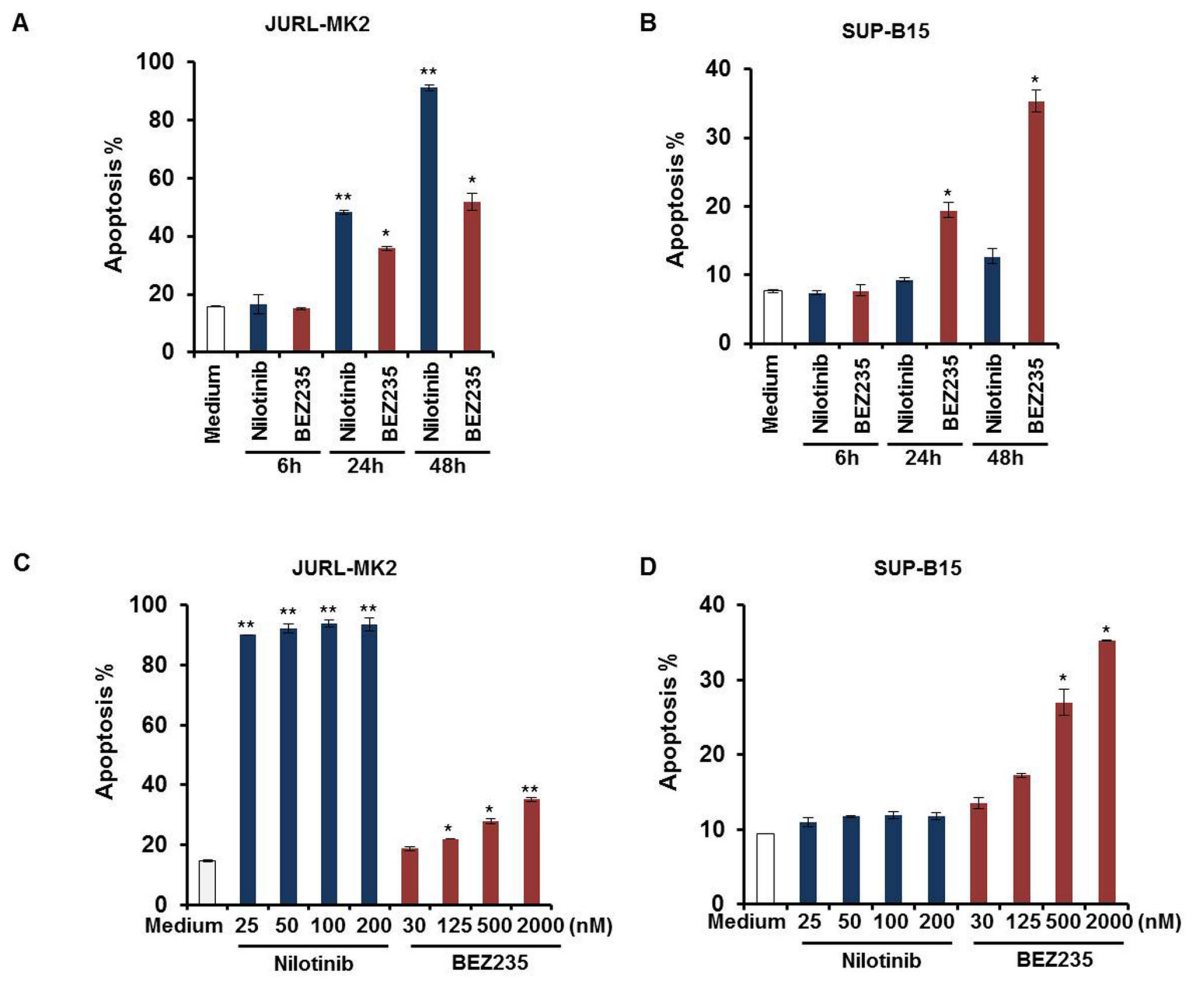
SUP-B15 cells are resistant to nilotinib treatment and both cell lines are sensitive to BEZ235. (A) (B) JURL-MK2 and SUP-B15 cells were treated with 100 nM nilotinib or 2 μM BEZ235 for 6 to 48 h. (C) (D) JURL-MK2 and SUP-B15 cells were incubated with different concentrations of nilotinib or BEZ235 as indicated for 48 h. Cell apoptosis was determined by annexin V/PI staining assay. Means ± SD (n=3) are shown. Statistical differences compared with the controls are given as *P<0.05, **P<0.01.

Ph+ cell lines JURL-MK2 and SUP-B15 were treated with TKI nilotinib or PI3K/mTOR inhibitor BEZ235 and apoptosis was tested by annexin-V/PI assay. Time-course and dose-response studies showed that JURL-MK2 cells were sensitive to the TKI nilotinib (Figure 1A and 1C). More than 80% of JURL-MK2 cells underwent apoptosis after 48 h (Figure 1A). In contrast, cell line SUP-B15 was not affected by nilotinib (Figure 1B and 1D). We have shown in a previous study, that nilotinib resistance was paralleled by the activation of the PI3K/AKT pathway [11]. Hence, we used the dual PI3K/mTOR inhibitor BEZ235 to find out whether constitutive PI3K activity was the cause of nilotinib resistance of SUP-B15 cells. Our data showed that BEZ235 induces apoptosis in nilotinib-sensitive (Figure 1A and 1C) and resistant cell lines (Figure 1B and 1D) in a time- and dose-dependent manner. The sensitivity to BEZ235 was similar in both cell lines. 2μM BEZ235 induced 35.2% apoptosis in JURL-MK2 (Figure 1C) and 35.3% apoptosis in SUP-B15 (Figure 1D) after 48h. 

### BEZ235 but not nilotinib induces G1 phase arrest in SUP-B15

**Figure 2:**
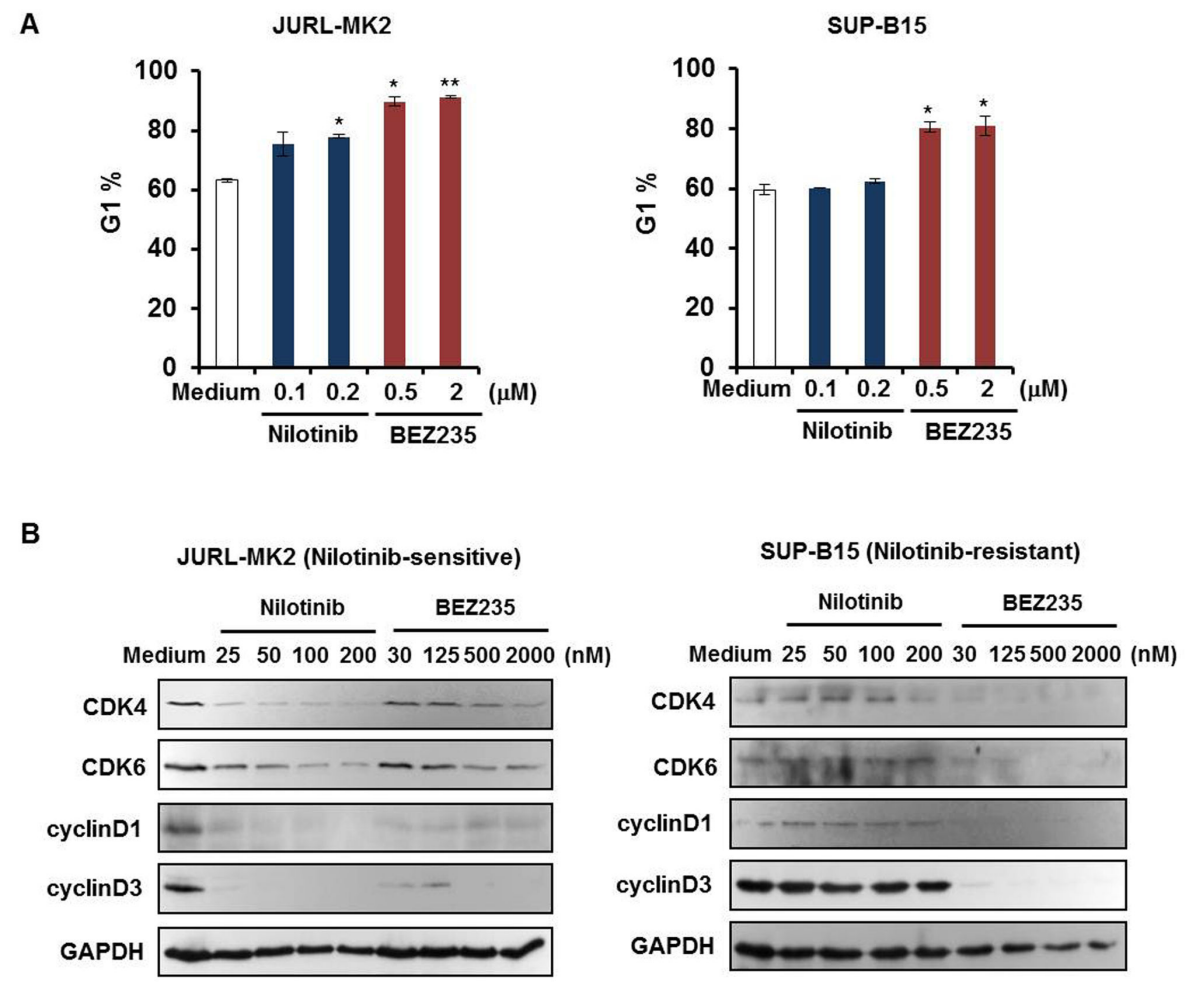
BEZ235 but not nilotinib induces G1 phase arrest in SUP-B15. (A) JURL-MK2 and SUP-B15 cells were treated with different concentrations of nilotinib or BEZ235 as indicated. After 24 h, cells were harvested, stained with PI and analyzed for cell cycle distribution by flow cytometry. The graph shows percentages of cells (± SD) in G1 phase. Data were representative of three independent experiments; *P<0.05 vs control, **P<0.01 vs control. (B) JURL-MK2 and SUP-B15 cells were treated with different concentrations of nilotinib or BEZ235 as indicated for 24 h. Total cellular lysates were analyzed by Western blot using the indicated antibodies.

In addition to BEZ235-induced apoptosis, cell cycle arrest was examined in JURL-MK2 and SUP-B15 cells using PI staining and flow cytometry analysis. Cells were treated with different concentrations of nilotinib or BEZ235. After 24 h, cells were harvested and the percentage of cells in G1 was measured. Both nilotinib and BEZ235 increased the cell fractions in G1 phase in JURL-MK2 cells (Figure 2A left). In SUP-B15 cells, only BEZ235 induced G1 phase arrest (Figure 2A right). To find out whether cell cycle arrest correlated with repression of proteins impacting G1 transition, we examined the effects of nilotinib and BEZ235 on the expression of G1 phase related cell cycle proteins (Figure 2B). The transition of G1 to S phase is a complex process involving cyclins and cyclin-dependent kinases (CDKs) [36]. CDK4, CDK6, cyclinD1 and cyclinD3 were all downregulated by nilotinib or BEZ235 in JURL-MK2 cells. In SUP-B15 cells, treatment with BEZ235 but not nilotinib suppressed these proteins. These results showed that BEZ235 not only induced apoptosis, but also promoted cell cycle arrest in JURL-MK2 and SUP-B15 cells by inhibiting related cell cycle proteins. In contrast, SUP-B15 cells proved resistant to the effects of nilotinib. Neither apoptotic cell death nor G1 phase arrest were induced by nilotinib in this cell line.

### GAB2 does not confer nilotinib resistance in SUP-B15 cells

**Figure 3:**
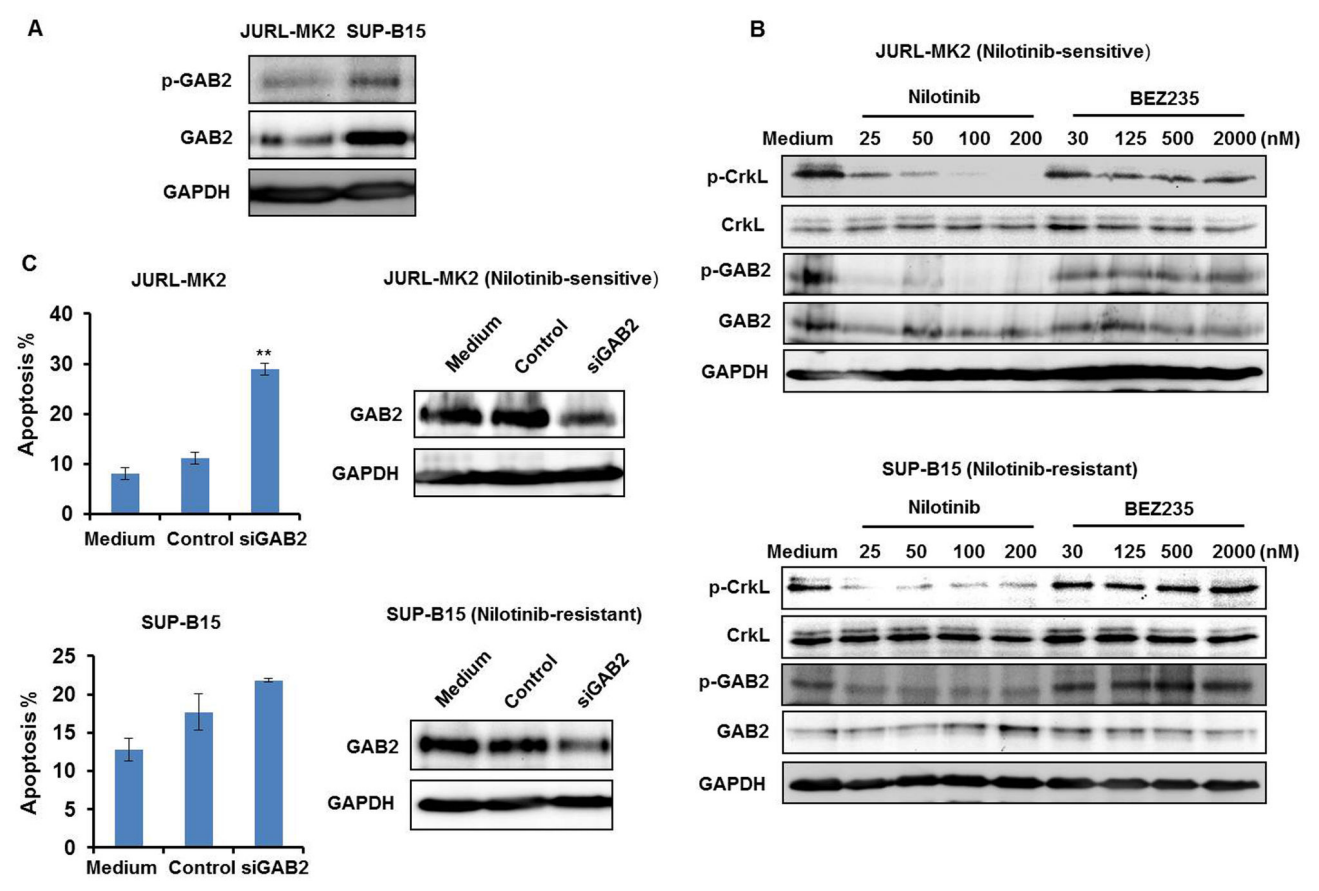
GAB2 does not confer nilotinib resistance in SUP-B15 cells. (A) Expression of GAB2 and p-GAB2 proteins in JURL-MK2 and SUP-B15 cells was analyzed by Western blot. GAPDH levels served as a loading control. (B) JURL-MK2 and SUP-B15 cells were treated with different concentrations of nilotinib or BEZ235 as indicated for 6 h. P-GAB2, GAB2, p-CrkL and CrkL expression were determined by Western blot. (C) GAB2 was knocked down in JURL-MK2 and SUP-B15 cells by siRNA inhibition as described in Materials and Methods. After 24 h, expression of GAB2 was analyzed by Western blot and cell apoptosis was determined by annexin V/PI staining assay. Means ± SD of two experiments are shown. **P<0.01 vs control.

JURL-MK2 and SUP-B15 are both Ph+ cell lines, containing unmutated BCR-ABL1 [11]. However, they reacted differently to nilotinib. In order to find out why one succumbed to treatment with TKI while the other survived, we focused on the BCR-ABL1 signaling network. Previously, our group found that BCR-ABL1-independent PI3K activation led to TKI resistance [11]. Therefore, we focused on members of the PI3K/AKT pathway to seek the cause for TKI resistance in cell line SUP-B15. Expression of GAB2 was analyzed first. Linking BCR-ABL1 and PI3K pathway, GAB2 serves as a critical amplifier in the BCR-ABL1 network [12]. Phosphorylated GAB2 Y452 is a PI3K recruitment site. It has been reported that GAB2 signaling protects CML cells from TKI inhibitor-induced cell death while GAB2 knockdown increases TKI sensitivity [12]. Based on these findings, we checked whether SUP-B15 cells expressed unusually high levels of GAB2, potentially causing nilotinib resistance. Western blot analysis confirmed significantly higher GAB2/p-GAB2 levels in SUP-B15 than in JURL-MK2 cells (Figure 3A). However, nilotinib impaired phosphorylation of GAB2 in both cell lines, demonstrating that the TKI-resistant cell line SUP-B15 was not unresponsive to nilotinib (Figure 3B). This conclusion was supported by the finding that nilotinib also induced dephosphorylation of the BCR-ABL1 target CrkL (Figure 3B).

Confirming the importance of GAB2 for BCR-ABL1-mediated cell survival, GAB2 knockdown induced apoptosis in JURL-MK2 cells (Figure 3C). However, this knockdown effect could not be observed in SUP-B15 cells (Figure 3C). Thus, inhibitor and knockdown experiments implied that in SUP-B15 cells the oncogenic signal responsible for the continuous stimulation of the PI3K and the reason for TKI unresponsiveness lay downstream of GAB2. 

### BEZ235 inhibition of mTOR pathway leads to block of MDM2 translation

**Figure 4:**
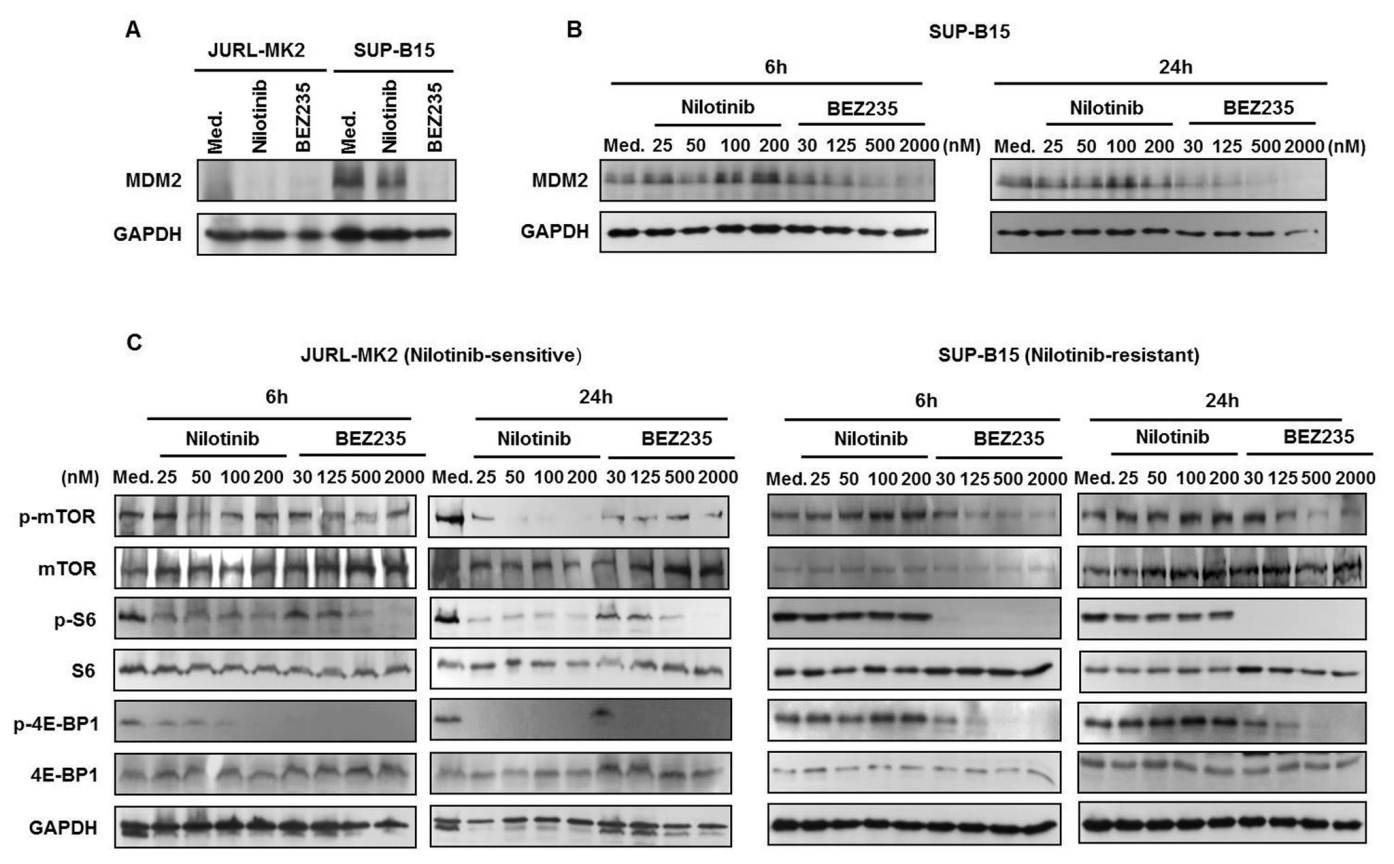
BEZ235 inhibition of mTOR pathway leads to block of MDM2 translation. (A) JURL-MK2 and SUP-B15 cells were treated with 100 nM nilotinib or 2 μM BEZ235 for 24 h. MDM2 protein levels were analyzed by Western blot. GAPDH levels served as loading control. (B) MDM2 protein expression in SUP-B15 cells was tested after 6 or 24 h with different concentrations of nilotinib and BEZ235. (C) JURL-MK2 and SUP-B15 cells were treated with various doses of nilotinib or BEZ235 as indicated for 6 and 24 h. mTOR and its downstream targets, S6 and 4E-BP1, together with their phosphorylation forms were determined by Western blot. Med., medium.

Since overexpression of GAB2 was not the reason for nilotinib resistance in SUP-B15, we dissected the downstream signaling chain to find the pathway members responsible for blockage of nilotinib-mediated cell death. To this end, we first compared the mRNA levels of PI3K/AKT pathway factors in JURL-MK2 and SUP-B15 cells by quantitative RT-PCR. Of all relevant genes, only the anti-apoptotic MDM2 showed elevated transcript levels in TKI-resistant SUP-B15 cells (Table 1). In accordance with the mRNA data, higher MDM2 protein expression was observed in SUP-B15 cells (Figure 4A). Interestingly, BEZ235 down-regulated MDM2 protein levels in a time- and dose-dependent manner in SUP-B15 cells, while nilotinib left these cells barely perturbed (Figure 4A/B). BEZ235 is a PI3K/mTOR inhibitor which induces cell cycle arrest and apoptosis in cancers [37,38]. In SUP-B15 cells, BEZ235 induced apoptosis accompanied by downregulation of MDM2 (Figure 1, Figures 4A and 4B). To find out how inhibition of PI3K/mTOR inhibited MDM2 expression, we performed Western blot analysis with lysates prepared from cells treated with BEZ235. Phosphorylation of PI3K downstream effector mTOR and its targets, 4E-BP1 and S6 was suppressed by BEZ235 and nilotinib in JURL-MK2 cells, whereas only BEZ235 was active in SUP-B15 cells (Figure 4C). It is known that phosphorylation of 4E-BP1 and S6 activates a cap-dependent mRNA translation [39,40]. Thus, our results suggested that BEZ235 abolished MDM2 expression at the translation level through blocking the translational machinery. Accordingly, BEZ235 triggered decrease of MDM2 protein, while sparing its transcription. After 24-hour BEZ235 (2 μM) treatment, MDM2 mRNA levels in SUP-B15 cells were unaffected (control: 1; BEZ235: 1.36). In summary, BEZ235 inhibition of mTOR pathway blocks MDM2 translation in SUP-B15 cells.

**Table 1:** Comparison of mRNA levels of PI3K/AKT pathway factors in JURL-MK2 and SUP-B15 cells using quantitative RT-PCR.

	PIK3CA	AKT	TSC1	TSC2	GβL	RAPTOR	RICTOR	mTOR	p70S6K	MDM2
JURL-MK2	1	1	1	1	1	1	1	1	1	1
SUP-B15	1.8	0.2	0.6	0.6	0.6	0.3	1.3	0.6	0.5	17.4

mRNA levels of different genes in JURL-MK2 were set as 1 and those in SUP-B15 were relative numbers compared to JURL-MK2. TSC1/2: tuberous sclerosis protein 1/2; GβL: G-protein beta-subunit-like; p70S6K: p70S6 kinase

### Combination of BEZ235 and nilotinib has a synergetic effect on apoptosis in SUP-B15 cells

**Figure 5:**
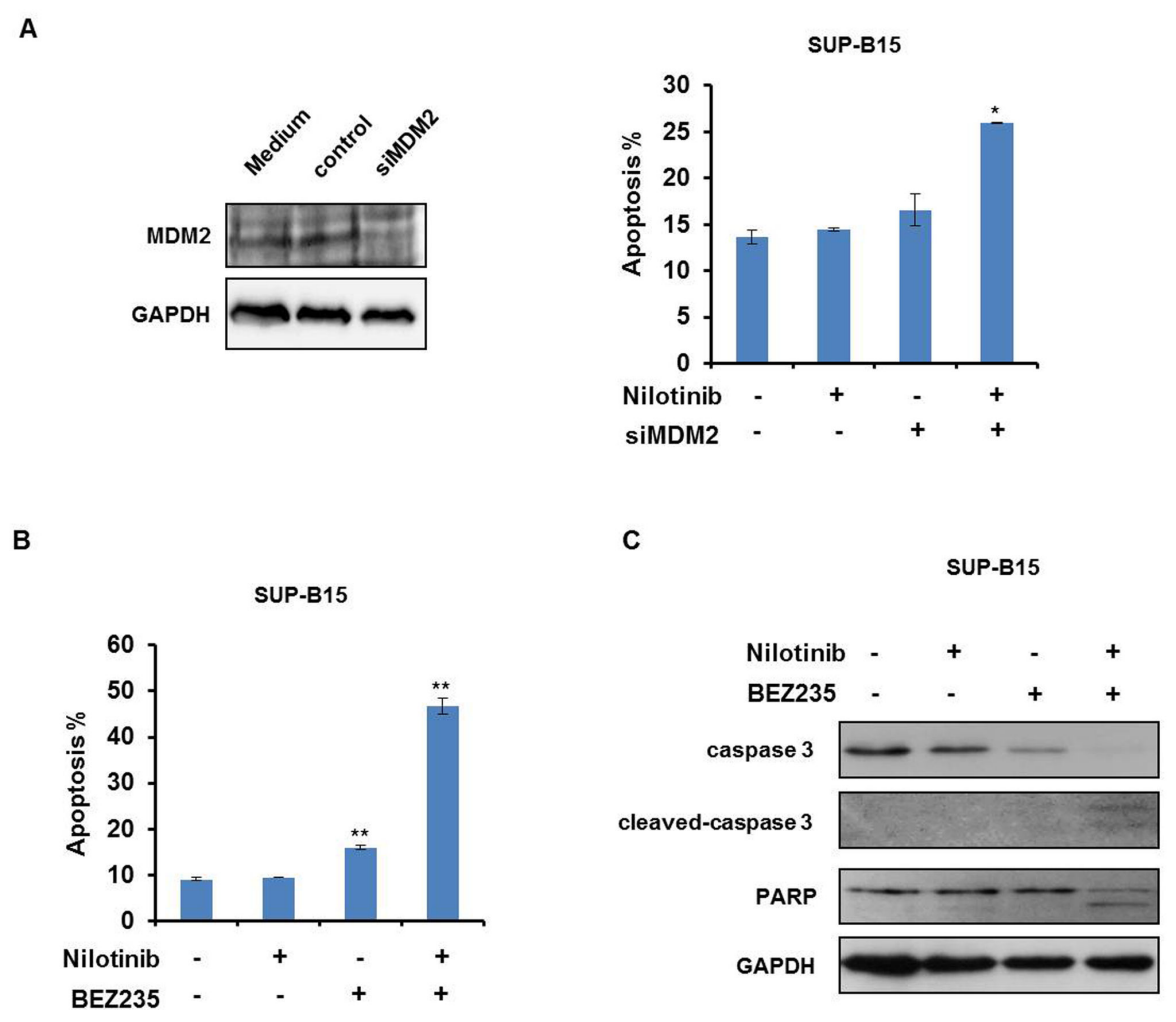
Combining nilotinib and BEZ235 synergizes in inducing apoptosis in SUP-B15 cells. (A) MDM2 knockdown in SUP-B15 cells was performed by siRNA, and MDM2 protein levels were examined by Western blot. SUP-B15 cells were treated with 200 nM nilotinib and/or MDM2 knockdown alone for 48 h. Apoptosis was analyzed by annexin V/PI staining assay. Means ± SD of three experiments are shown. *P<0.05 vs control. (B) SUP-B15 cells were treated with nilotinib (200 nM) and/or BEZ235 (2μM) for 24 h. Apoptotic cell death was determined by annexin V/PI staining assay. Means ± SD of three experiments are shown. **P<0.01 vs control. (C) SUP-B15 cells were treated with nilotinib (200 nM) or BEZ235 (2 μM) either alone or in combination for 24 h. Cell lysates were subjected to Western blot analysis with antibodies against caspase 3 and PARP as indicated. For equal protein loading GAPDH protein levels are shown.

**Figure 6:**
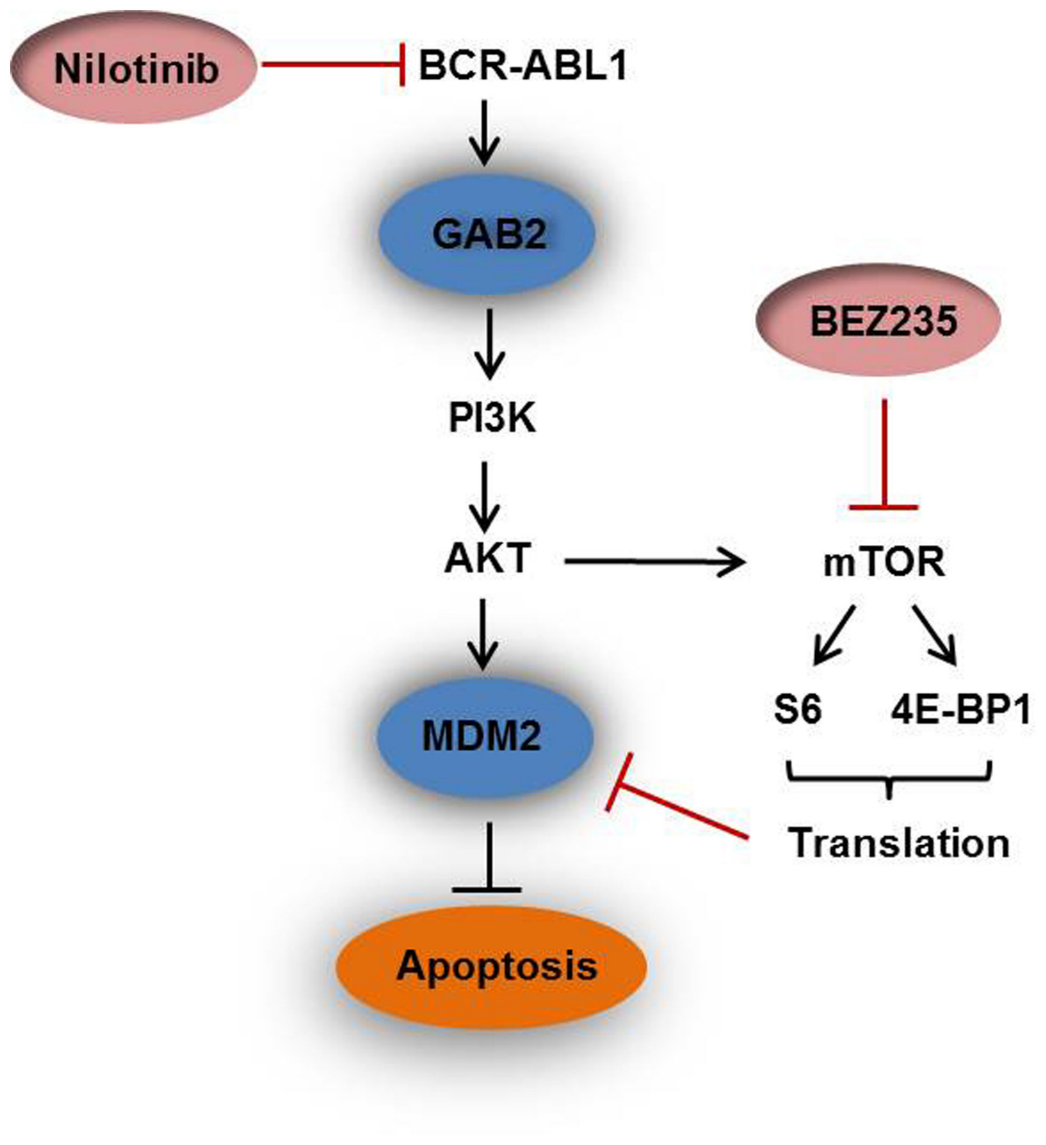
Scheme explaining the synergistic effects of combining nilotinib and BEZ235. BEZ235 could abrogate MDM2 protein expression in SUP-B15 cells, suppressing the translational machinery evidenced by dephosphorylation of S6 and 4E-BP1. Inhibition of the BCR-ABL1 by nilotinib and simultaneous down-regulation of the anti-apoptotic protein MDM2 by BEZ235 synergizes in inducing apoptosis in SUP-B15 cells.

Based on the finding that BEZ235 but not nilotinib reduced MDM2 protein expression in SUP-B15 cells we speculated whether MDM2 overexpression was the cause for nilotinib resistance. To this end, we performed MDM2 knockdown experiments. Suppression of MDM2 alone barely induced apoptosis in the nilotinib-resistant cell line SUP-B15 (Figure 5A). However, MDM2 knockdown sensitized the cell line to nilotinib confirming that the high MDM2 expression levels were at least partly responsible for TKI resistance (Figure 5A). Like MDM2 knockdown, BEZ235 also rendered SUP-B15 cells sensitive to nilotinib (Figure 5B), explicable by the finding that BEZ235 treatment promoted downregulation of MDM2 (Figure 4A and 4B). Induction of apoptotic cell death was further confirmed by Western blot analysis showing cleavage of caspase 3 and consequently PARP cleavage in SUP-B15 cells treated with a combination of nilotinib and BEZ235 (Figure 5C). Collectively, these data showed that inhibition of BCR-ABL1 by nilotinib and simultaneous down-regulation of the anti-apoptotic protein MDM2 by BEZ235 synergized in inducing apoptosis in SUP-B15 cells (Figure 6).

## Discussion

The BCR-ABL1 oncogene contributes to the development of CML clones. BCR-ABL1 does not only occur in CML, since 20-30% of ALL are also Ph+. TKIs are effective for CML therapy. In a previous study, we tested 19 Ph+ CML and ALL cell lines for TKI responsiveness. 5/19 (KCL-22, NALM-1, SD-1, SUP-B15 and MHH-TALL-1) were TKI-resistant, although none showed mutation in the kinase domain of BCR-ABL1. Resistance was also not independent of BCR-ABL1 size variance. All three BCR-ABL fusion proteins (p230, p210 and p190) exhibit deregulated tyrosine kinase activity compared with the native ABL protein [41]. Resistance occurred in p210 BCR-ABL1 positive CML cell lines as well as p190 BCR-ABL1 positive ALL cell lines [11]. We found that the constitutive and BCR-ABL1-independent activation of the PI3K/AKT pathway was a common feature of all resistant cell lines [11]. In this study, we set out to dissect the BCR-ABL1-PI3K/AKT/mTOR pathway to further investigate TKI resistance mechanisms and sensitization of resistant tumor cells to TKI treatment.

GAB2 is a scaffold protein, linking plasma membrane receptor signaling (including receptor tyrosine kinases) with downstream effectors. GAB2 acts as signal transducer downstream of BCR-ABL1, thus contributing to TKI resistance in CML cells [12]. GAB2 plays a prominent role in leukemia, breast and ovarian cancer and melanoma [42]. GAB2-positive myeloid cells are more frequent in CML than in healthy controls (unaffected hematopoiesis) and their number increases markedly from chronic to accelerated phases and onto blast crisis [43]. We confirmed that BCR-ABL1-activated GAB2 was crucial to survival of CML cells and that knockdown of GAB2 led to apoptosis in the TKI-sensitive CML cell line JURL-MK2 (Figure 3C). This result is consistent with the previous finding that GAB2 knockdown affects CML viability and proliferation [44]. The TKI-resistant ALL cell line SUP-B15 expressed higher levels of GAB2 than JURL-MK2 cells, suggesting that its overexpression might underlie TKI resistance in this cell line (Figure 3A). However, nilotinib efficiently inhibited GAB2 phosphorylation, while GAB2 knockdown did not affect the viability of SUP-B15 cells (Figure 3B and 3C). This finding is consistent with the previous report that GAB2 is more critical for myeloid than lymphoid transformation by BCR-ABL1 [45] and indicates that nilotinib resistance resulted from oncogenic stimuli targeting the PI3K/AKT pathway downstream of GAB2.

We compared expression of the members of PI3K/AKT signaling pathway and found that of these only MDM2 was overexpressed in the resistant cell line SUP-B15 (Table 1 and Figure 4A), consistent with previous reports in pediatric ALL cells [21,46]. Here, we additionally show that MDM2 is related to TKI-resistance. MDM2 is an E3 ubiquitin ligase which limits the expression of p53 [26]. To test whether the alternative expression of MDM2 was the cause for TKI resistance, we knocked down MDM2 in SUP-B15 cells. The knockdown of MDM2 alone induced apoptosis in just a small percentage of cells (Figure 5A). However, partial MDM2 deletion led to a recovery of sensitivity to TKI nilotinib (Figure 5A), confirming that the high expression of MDM2 was indeed important for TKI resistance of SUP-B15 cells. 

MDM2 is a protein with a very short turnover time [47]. Therefore, inhibition of the translational machinery should lead to a rapid downregulation of this protein. BEZ235 is a PI3K/mTOR dual inhibitor and repression of mTOR downstream targets 4E-BP1 and S6 which blocks translation [32]. Phosphorylation of the key translation regulators 4E-BP1 and S6 generally controls the cap-dependent translation of mRNAs [39,48]. Accordingly, BEZ235 led to a time- and dose-dependent dephosphorylation of mTOR and its two targets S6 and 4E-BP1, and induced degradation of MDM2 (Figure 4). BEZ235 treated SUP-B15 cells regained TKI-responsiveness, as combined treatments with both drugs very efficiently induced apoptosis (Figure 5B and 5C). BEZ235 was even more efficient than MDM2 knockdown to reconstitute TKI responsiveness of SUP-B15 cells, which might be due to incomplete MDM2 knockdown (Figure 5A). Alternatively, BEZ235 might, in addition to MDM2, also affect other BCR-ABL1 targets. Supporting this view, we found that BEZ235 induced dephosphorylation of ERK1/2 (Figure S1). Notwithstanding the potentially more indirect effects of BEZ235, our knockdown and inhibitor experiments provide solid evidence that MDM2 overexpression was pivotal for TKI resistance in SUP-B15 cells. Inhibition of PI3K/mTOR pathway helped to regain TKI responsiveness. These results are consistent with the finding that compared with targeting individual components of the PI3K/AKT signaling module alone, inhibition of this pathway at multiple levels using dual-specificity inhibitors, or combining specific pathway inhibitors with classic regimens may be more effective for leukemia therapy [49]. 

We previously reported that 5/19 Ph+ CML/AML (acute myeloid leukemia) cell lines (KCL-22, NALM-1, SD-1, SUP-B15 and MHH-TALL-1) were TKI-resistant and that none showed mutations in the kinase domain of BCR-ABL1. We identified the PI3Kα E545G mutation in cell line KCl-22 [11] and the mutationally inactivated PI3K-inhibitor PTEN (Phosphatase and tensin homolog) in the MHH-TALL-1 cell line (Figure S2) as potential causes for constitutive activity of the PI3K/AKT pathway conferring TKI resistance to these cells. In this study, we found that overexpression of the anti-apoptotic protein MDM2 contributes to TKI resistance and that suppression of MDM2 restores sensitivity to TKI. 

In conclusion, we used Ph+ cell lines JURL-MK2 and SUP-B15 to investigate mechanisms of TKI resistance and sensitization. TKI nilotinib effectively induced cell apoptosis in JURL-MK2, but not in SUP-B15 cells. Inhibition of BCR-ABL1 by nilotinib and simultaneous down-regulation of the anti-apoptotic protein MDM2 by BEZ235 synergized in inducing apoptosis in SUP-B15 cells (Figure 6). BEZ235 operated via blocking the translational machinery as evidenced by dephosphorylation of S6 and 4E-BP1. Taken together, these results suggest that MDM2 may be a therapeutic target to increase TKI-mediated apoptosis and that combining PI3K/mTOR inhibitor and TKI may prove an effective novel therapeutic strategy in TKI-resistant BCR-ABL1 positive leukemia.

## Supporting Information

Figure S1 **BEZ235 inhibits phosphorylation of BCR-ABL1 downstream signaling molecule ERK but not STAT5 in SUP-B15 cells.** (A) SUP-B15 cells were respectively treated with 100 nM Imatinib, 100 nM nilotinib, 100 nM Everolimus or 2 μM BEZ235 for 24 h. p-ERK and ERK protein levels were analyzed by Western blot. (B) P-STAT5 and STAT5 protein expression in SUP-B15 cells were tested by Western blot after 24 h treatment with different concentrations of nilotinib and BEZ235.
(TIF)

Figure S2 **Genomic silencing of PTEN in MHH-TALL-1.** (A) Fluorescence in situ hybridization analysis showed intact IKZF1 (left) but loss of the genomic region hosting PTEN (right) covering much of BAC clone RPM1-383D9 (red) and all of the neighboring clone RPMI-879E1. Conventional cytogenetic analysis showed no evidence of genomic loss, hence indicating focal microdeletion of the centromeric part of chromosome band 10q23.31 (not shown). (B) Genomic PCR confirmed the hemizygous loss of PTEN in cell line MHH-TALL1. (C) The remaining PTEN allele had a one base pair insertion leading to a premature stop after amino acid 241, evidenced by sequencing of the RT-PCR product of cell line MHH-TALL1. (D) Cell line MHH-TALL1 did not express the PTEN protein according to Western blot analysis. T, T-cell; B, B-cell; M, myeloid; r, resistant; s, sensitive; n.d., not done.
(TIF)
