# Supplementary figures and images for: Inhibition of PI3K/mTOR Overcomes Nilotinib Resistance in BCR-ABL1 Positive Leukemia Cells through Translational Down-Regulation of MDM2
Source: PLoS One. 2013 Dec 11;8(12):e83510. doi: 10.1371/journal.pone.0083510 (PMC3859659; doi:10.1371/journal.pone.0083510)

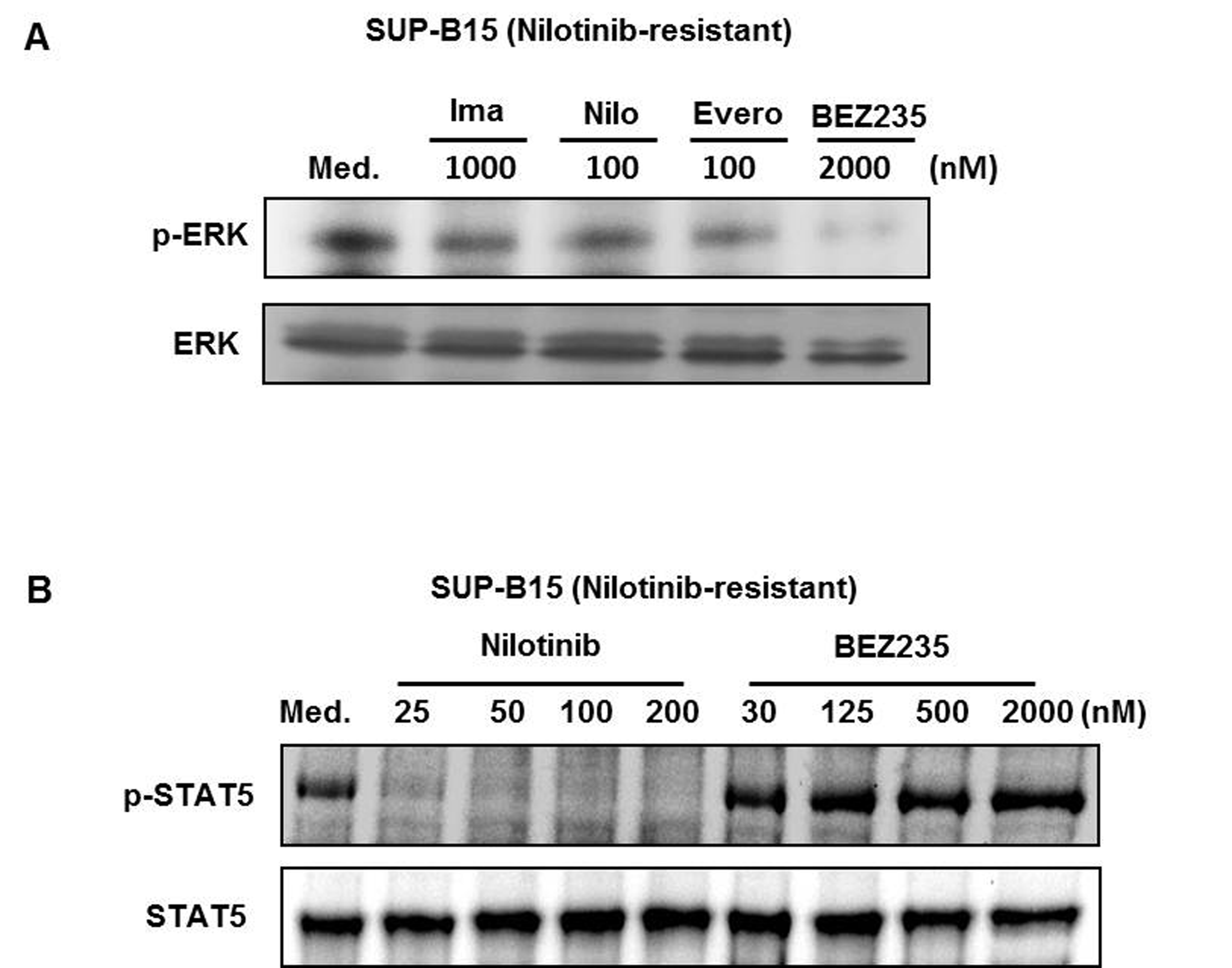

Supplement: Figure S1 — BEZ235 inhibits phosphorylation of BCR-ABL1 downstream signaling molecule ERK but not STAT5 in SUP-B15 cells. (A) SUP-B15 cells were respectively treated with 100 nM Imatinib, 100 nM nilotinib, 100 nM Everolimus or 2 μM BEZ235 for 24 h. p-ERK and ERK protein levels were analyzed by Western blot. (B) P-STAT5 and STAT5 protein expression in SUP-B15 cells were tested by Western blot after 24 h treatment with different concentrations of nilotinib and BEZ235. (TIF) [file pone.0083510.s001.tif]

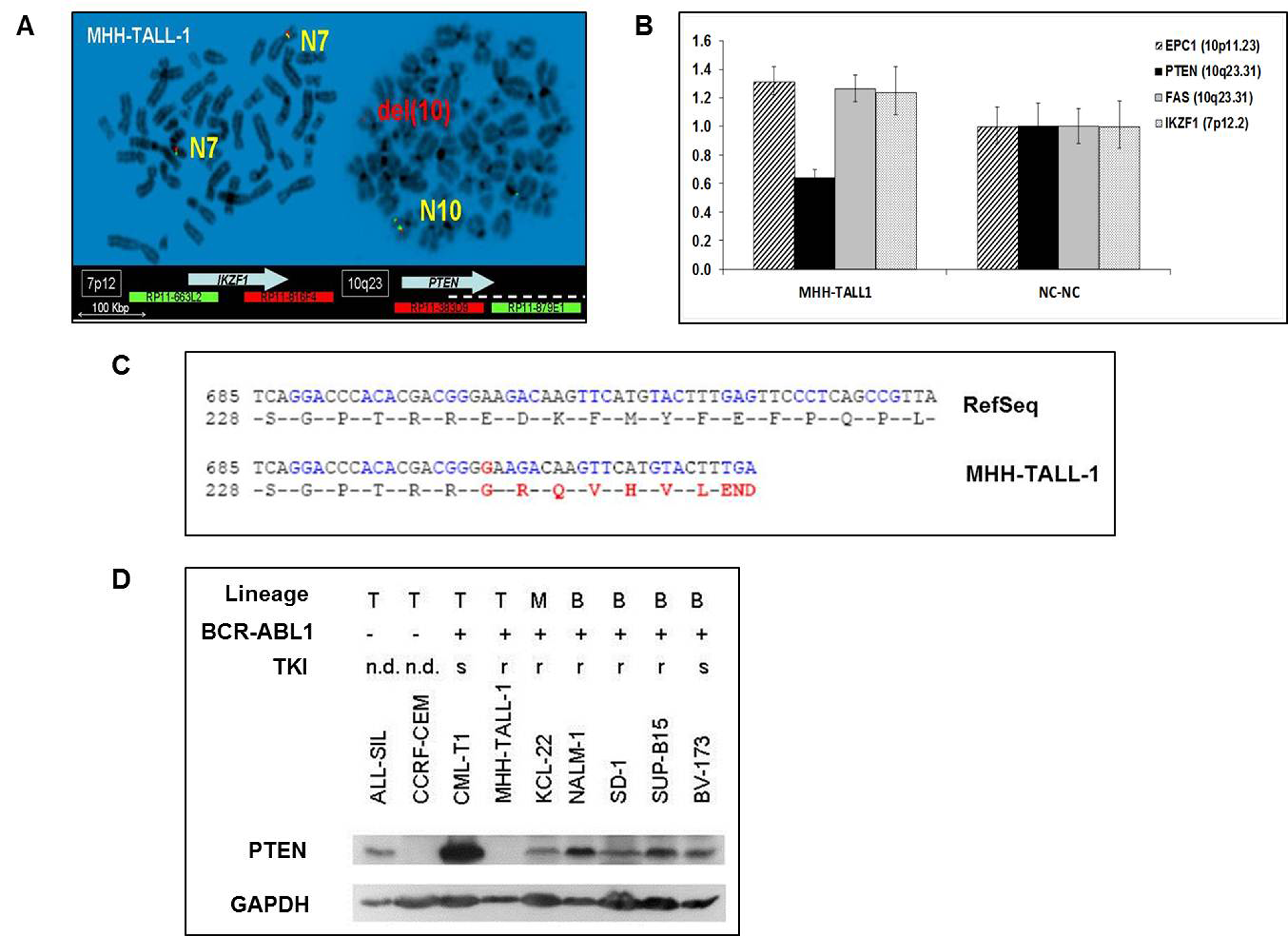

Supplement: Figure S2 — Genomic silencing of PTEN in MHH-TALL-1. (A) Fluorescence in situ hybridization analysis showed intact IKZF1 (left) but loss of the genomic region hosting PTEN (right) covering much of BAC clone RPM1-383D9 (red) and all of the neighboring clone RPMI-879E1. Conventional cytogenetic analysis showed no evidence of genomic loss, hence indicating focal microdeletion of the centromeric part of chromosome band 10q23.31 (not shown). (B) Genomic PCR confirmed the hemizygous loss of PTEN in cell line MHH-TALL1. (C) The remaining PTEN allele had a one base pair insertion leading to a premature stop after amino acid 241, evidenced by sequencing of the RT-PCR product of cell line MHH-TALL1. (D) Cell line MHH-TALL1 did not express the PTEN protein according to Western blot analysis. T, T-cell; B, B-cell; M, myeloid; r, resistant; s, sensitive; n.d., not done. (TIF) [file pone.0083510.s002.tif]
